# A Comprehensive Review of Plant and Microbial Natural Compounds as Sources of Potential *Helicobacter pylori*-Inhibiting Agents

**DOI:** 10.3390/biotech14040094

**Published:** 2025-11-26

**Authors:** Srichandan Padhi, Swati Sharma, Puja Sarkar, Marco Masi, Alessio Cimmino, Amit Kumar Rai

**Affiliations:** 1 University Centre for Research and Development, Chandigarh University, Mohali 140413, India; 2 Advanced Centre of Research and Innovation, Chandigarh Group of Colleges Jhanjeri, Mohali 140307, India; swatishimla12@gmail.com; 3 Food and Nutrition Biotechnology, BRIC-National Agri-Food and Biomanufacturing Institute (NABI), Mohali 140306, India; pujas8981@gmail.com; 4 Department of Chemical Sciences, University of Naples Federico II, Complesso Universitario Monte S. Angelo, Via Cintia, 80126 Naples, Italy; alessio.cimmino@unina.it; 5 Department of Botany, University of Delhi, New Delhi 110007, India

**Keywords:** natural products, *Helicobacter pylori*, antimicrobials, gastric pathogenesis

## Abstract

*Helicobacter pylori*, the gastric pathogen which colonizes the gastric mucosa of more than half of the world’s population, is considered a risk factor for peptic ulcers and is epidemiologically associated with gastric cancer. Antimicrobial eradication of this pathogen has now become a central concern because of its growing resistance to frontline antibiotics such as clarithromycin and metronidazole. Moreover, these antibiotics can have adverse effects on the normal human gut flora and can lead to several health complications. Most times, the antibiotic doses become intolerable to the elderly population and they reject the therapy. This has impelled us to think about alternate effective and safe antimicrobials which can replace antibiotic usage or may reduce their dosage when used together with the antibiotics. Plant and microbial natural products, in view of this, offer an excellent source of novel and potential antimicrobial agents. Herein, we review anti-*H. pylori* natural compounds from diverse plant and microbial sources and highlight their role in the management of *H. pylori* infection.

## 1. Introduction

Plants and microorganisms are well-known producers of a diverse array of therapeutically important chemical compounds called secondary metabolites. To date, myriads of natural compounds have been documented, of which plant and microbial secondary metabolites contribute a major part, and more than 50% of FDA-approved drugs have directly or indirectly been derived from them [1]. However, such metabolites still appear to be an inexhaustible source of new and effective bioactive agents or drugs. Plants produce these compounds as a part of defense against invading pathogenic microbes, pests and herbivores. Microorganisms, however, in the late growth phase, synthesize these chemicals when one or more of the components in the nutrient medium is run down or in response to environmental stress.

*Helicobacter pylori*, a human pathogenic bacterium which infects and colonizes the human stomach, is the main cause of chronic gastritis and gastric inflammation. In most cases, the infection remains asymptomatic; only a lesser portion develops into severe or very severe clinical outcomes. However, it has been recently established that the majority of gastric adenocarcinoma and gastric mucosa-associated lymphoid tissue (MALT) lymphoma cases are due to infection with *H. pylori* [2,3]. Over two thirds of the world’s population is infected with *H. pylori.* The bacterium is attained during childhood and can continue to colonize thereafter if left untreated. Major virulence factors implicated in the infection process include cytotoxin-associated gene A (CagA) and vacuolating cytotoxin A (VacA). In addition, this bacterium escapes the action of antibiotics and also the host immune response by forming biofilm (aggregated free-floating planktonic cells sheathe themselves in an extracellular matrix composed of polysaccharides, DNA and proteins). The clinician-recommended treatment for the last twenty years includes a triple therapy consisting of antibiotics such as clarithromycin and amoxicillin or metronidazole along with a proton pump inhibitor (PPI) or ranitidine bismuth citrate; nonetheless, overuse of such medications is leading to the development of drug resistance in *H. pylori* and causes treatment failure in more than 20% of patients [4]. Moreover, such therapies are expensive and have been associated with obvious side effects similar to those observed with other drugs used for cardiovascular problems and allergies [5]. Given the advantages of plant and microbial secondary metabolites in terms of potency and safety over synthetic or industrially produced drugs [6], natural anti-*H. pylori* agents with novel action mechanisms are urgently needed in medicine. This review focuses on a selection of anti-*H. pylori* natural compounds derived from plant extracts and microbial culture filtrates and groups them as per their mode of action.

## 2. *Helicobacter pylori*: History of Infection and Associated Pathologies

*H. pylori* has been designated as a Class I carcinogen by the World Health Organization (WHO), and its rising incidences and prevalence globally are a serious concern. It was in 1983 that two researchers, Marshall and Warren, at the Royal Perth Hospital, Australia, reported an unidentified curved bacillus in the gastric epithelia in patients with chronic gastritis. A year later, in 1984, Marshall and Warren published another report describing the role of that bacterium in peptic ulceration and identified it as *H. pylori*. In 2005, both scientists were awarded the Nobel Prize in Physiology or Medicine. Until then, there had been long-standing speculation that psychological stress and lifestyle factors were the main causes of gastritis and peptic ulcers. Now, it is obvious that *H. pylori* infections are very common and have affected a major part of the world’s human population. Over time, it has become evident that *H. pylori* is present in all human races, on all continents, and can be attributed to a number of dissimilar factors including geographical parameters and socioeconomic practices [7].

Gastric colonization by *H. pylori* is reported to provoke major pathologies which include chronic gastritis, peptic ulcers, MALT lymphoma and gastric adenocarcinoma [8]. The determination of risks, from simple infection to the development of clinical diseases, can be correlated to the patterns and severity of the colonization and depends on several factors including those relating to the bacterium, the host and the environment. *H. pylori* colonization is always associated with the infiltration of gastric mucosa (both in the antrum and in the corpus) with mononuclear and neutrophilic cells. The acute phase of infection when a person has accidentally consumed *H. pylori* or come into contact with contaminated material may be associated with non-specific dyspeptic symptoms including nausea and vomiting, pangastritis and hypochlorhydria, which may last for months. On the other hand, in the case of persistent colonization, a close correlation exists between the level of acid secretion in the stomach and gastritis distribution. This may be due to two counteractive effects: one results from the effects of acid secretion on the bacteria density, whereas the other results from the effects of bacterial growth and coupled gastric inflammation on the secretion and regulation of gastric acid. Peptic ulcers, which is a term that collectively refers to gastric and duodenal ulcers, are mucosal defects that penetrate through the muscularis mucosa. Gastric ulcers are found mostly at the shift from corpus to antrum mucosa (the zone with decreased acid output), while duodenal ulcers are found in the duodenal bulb, which has the most exposure to gastric acid. Both ulcers are linked firmly to *H. pylori* colonization. Initial reports of peptic ulcers from around the world have estimated that 85% of gastric and 95% of duodenal ulcers were due to *H. pylori* colonization and inflammation, of which 50% of cases demonstrated ulcer recurrences within one year, even after eradication of the bacterium. Bleeding, formation of stricture and perforation are so far the known complications of peptic ulcer disease. Recent data showed that the risk of the development of a peptic ulcer in the presence of *H. pylori* is about 10–12% [9,10].

**Figure 1:**
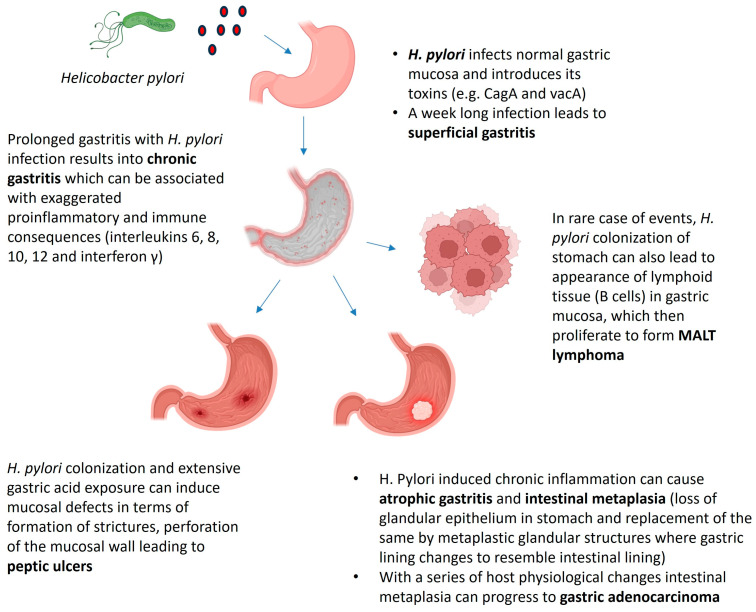
Illustration of host pathologies associated with *H. pylori* infection.

*H. pylori*-induced chronic inflammation can sooner or later affect the normal architecture of gastric mucosa with loss of the gastric glandular epithelium and replacement of either fibrosis or metaplastic glandular structures [11]. This is the process of atrophic gastritis and intestinal metaplasia, and has affected over half of the *H. pylori*-colonized population. In addition, areas of inflammation extend with time with no specific symptoms leading to increased risks of gastric cancer, the fourth most common cancer in the world which affects a large part of the world. Thus, gastric cancer is considered a major pathology of *H. pylori* colonization and its prevention has remained a major focus of research in recent times. MALT lymphoma is another serious pathologic condition characterized by the appearance of lymphoid tissue in the gastric mucosa in response to *H. pylori* colonization. Eventually, however, in rare events, this tissue may produce a population of monoclonal B cells which then gradually proliferate to MALT lymphoma. Data from several cohort studies suggest that the majority of patients with MALT lymphoma are *H. pylori* positive and that the presence of *H. pylori* infection increases the risks of gastric MALT lymphoma development [12]. In addition, *H. pylori* infection of the stomach elicits exaggerated inflammatory and immune reactions where pro-inflammatory interleukins IL-6 and IL-8 appear in the early cascade [13,14]. The infection also triggers other interleukins, IL-10, IL-12 and interferon (IFN)-γ, in a Th1-type gastric immune response [15]. The precise action mechanism through which the infection proceeds to severe clinical outcomes has not yet been properly established; nevertheless, it is a cumulative effect of several elements which include host and environmental parameters and, more importantly, bacterial virulence factors. An illustration displaying human pathologies associated with *H. pylori* infection is presented in Figure 1.

## 3. Antibiotic Resistance in *H. pylori*: Paving the Way for Discovery of New Antibacterials

Antibiotic resistance in *H. pylori* is a serious concern worldwide. Three major patterns, single-drug resistance (SDR), multidrug resistance (MDR) and heteroresistance (HR, population-wide variable resistance to one or several drugs), can be seen in *H. pylori*. All these patterns are overlapping and related, and are believed to impose definite clinical issues. In the past, no monotherapeutic treatment strategy has succeeded in achieving adequate efficacy. A few antibiotics, including amoxicillin, clarithromycin, metronidazole, tetracycline, rifabutin and levofloxacin, are being used in combination therapies that constitute two or three of them along with a bismuth compound or an acid inhibitor for effective *H. pylori* eradication [16]. Their extensive uses in the general population and the ability of bacterial species to adapt have led to the development of primary antibiotic resistance. Over the past decades, escalating antibiotic resistance in *H. pylori* has been witnessed across the global population despite the disparities in infection rates and profiles in diverse geographical regions. Most of the recommended first-line treatment regimens continue to suffer failures in about 10–30% of patients, which leads to a therapeutic predicament among patients after consecutive unsuccessful treatments [17,18]. In general, the emergence and spread of such resistance is leading to a significant decrease in treatment efficacy while at the same time potentially causing increased risks of severe pathophysiological complications. In view of this, the WHO has listed drug-resistant *H. pylori* (clarithromycin-resistant *H. pylori* in particular) on the priority (high) list of threats for which new and effective antibiotics are an urgent need (WHO news, 27 February 2017).

## 4. Bioactive Natural Products: The Improvement over Pharmaceutical Drugs

Traditional medicines have been serving us since the inception of human civilization. Despite this, we know far less about them than we do about conventional synthetic drugs. However, in the modern world, many have turned towards the use of natural medicines because of their benefits in healing ailments including anxiety and depression, cardiovascular diseases, diabetes, cancer and several other infectious diseases. It may not be wise to say that just because they are natural, they are always safe and non-toxic. The toxic effects reported from any source might have been attributable to inherent chemical structures, the concentration or doses administered or exposure duration [19].

In the 20th century, crude or semi-pure natural extracts, including those from plants and microbes, were the only measures available to treat human ailments. The years that followed witnessed a complete revolution of our thought process regarding the uses of drugs. The physiological effects of natural extracts in the body are facilitated by the interactions of their chemical components with biomolecules, including protein and nucleic acid receptors in the body (otherwise known as the receptor theory of drug action) [19]. This idea led to the establishment of a new era, and pure compounds purified and isolated from natural extracts rather than crude formulations turned into standard treatments. In due course, various natural compounds from plants and microorganisms were isolated and their bioactive effects characterized. Widely known examples include morphine (a natural compound from opium, used as a painkiller) and digoxin (isolated from the flower of *Digitalis lanata* Ehrh., used as a heart stimulant).

The biosynthesis process of natural products engages through repeated interaction with several modulating proteins and enzymes. This binding ability is believed to be an essential prerequisite for effective drug discovery [20]. On the other hand, pharmaceutical drugs are designed by chemical modification of existing drugs and are meant to bring out a specific reaction; however, they are often associated with obvious or adverse side effects considered to be risks compared to their primary beneficial effects [21,22]. This is how natural products complement synthetic drug molecules and invigorate pharmaceutical companies to reorient their efforts and resources towards natural product-based therapeutics discovery.

## 5. Plant and Microbial Natural Products as *H. pylori* Inhibitors

The potential and usefulness of plant and microbial natural products in traditional medicine are substantially known. As discussed in the earlier section, certain bioactive compounds or metabolites which are the intermediates or products of metabolism are responsible for physiological effects on the human body. These metabolites are highly diverse in terms of structure, function and biosynthesis, and are typically classified (according to their chemical structures) into terpenoids (built from isoprene units), phenolics (containing a phenol group), alkaloids (non-protein nitrogen-containing compounds) and polyketides (acetyl-CoA-derived compounds). Moreover, they have been associated with specific biological activities, and have become important clues in drug discovery. In this section, the bioactive significance of such metabolites as potent inhibitors of *H. pylori* is discussed and they are grouped as per their mechanism of action (specific or unspecific).

### 5.1. Inhibitors of H. pylori with a Specific Mechanism of Action

#### 5.1.1. Inhibitors of *H. pylori* Cytotoxins

The CagA and VacA proteins have thus far been reported to be the major virulence proteins of *H. pylori* and both are associated with an increased risk of gastro-duodenal disorders [23]. CagA is an immunodominant protein produced by most of the virulent strains of *H. pylori*. It is translocated to the host cell cytoplasm by a type IV secretion system during infection and is phosphorylated by the host Src and Alb kinases at a specific region called the Glu-Pro-Ile-Tyr-Ala (EPIYA) motif which is responsible for the biological activity of the pathogen. In addition, CagA interacts with multiple host receptor molecules and causes imbalance in the homeostasis process of the gastric epithelia, leading to induction of chronic inflammation and gastric carcinogenesis [24]. VacA is a secreted pore-forming cytotoxin and the gene for it is harbored by all *H. pylori* strains. VacA induces cellular autophagy and interrupts the lysosomal trafficking process. This, in turn, leads to accretion of abnormal autophagosomes and formation of vaculoles inside the cells, which promotes the immunotolerance and intracellular survivability of the bacterium [25]. Several natural compounds demonstrating inhibition or down-regulation of CagA and VacA in vitro were shown to have arrested the growth of different *H. pylori* strains. Examples include the plant metabolites evodiamine [26], hesperetin [27] and β-caryophyllene [28]. Additionally, two terpenes isolated from the plant *Nigella sativa* L., thymol [29] and thymoquinone [30], have displayed in silico inhibition of both CagA and VacA using molecular docking; nonetheless, further validation of this in vitro and in vivo is lacking.

#### 5.1.2. Inhibitors of *H. pylori* Urease

The survival of *H. pylori* in the harsh, acidic gastric environment is reported to be mediated by the urease enzyme, which utilizes the urea present in the stomach and produces ammonia that acts as a receptor for the H^+^ ions to create a neutral pH environment. On the other hand, urea and ammonia weaken the mucosal layer and form lesions on the internal mucosal lining of the stomach. Urease also stimulates the host immune system by inducing the activation of monocytes and neutrophils, which ultimately results in inflammatory lesions. To date, *H. pylori* urease is the most explored target for inhibitor design and anti-*H. pylori* drug discovery [31]. The secondary metabolites isolated and identified from different plant parts, including methyl rosmarinate [32], sanguinarine [33], terpineol [34] and zerumbone [35], have displayed promising inhibition of urease in vitro. In addition, a few phenolic compounds of plant origin, such as pyrocatechol, chlorogenic acid [36] and diosmin [37], have displayed binding interactions and inhibition of urease in silico.

#### 5.1.3. Inhibitors of *H. pylori* Homeostatic Stress Regulator A (HrsA)

The *H. pylori* genome carries genes that encode HrsA, an important and essential protein for microbial viability, which also acts as a global homeostatic regulator orchestrating metabolic functions and virulence, subject to nutrient availability, and arbitrating the responses to oxidative stress. In addition, HrsA modulates expression of several other genes involved in vital functions of host cells, including transcription, translation, redox homeostasis and metabolism of nitrogen [38,39]. A small number of natural products including a few flavones such as chrysin [39], apigenin [40] and kaempferol [41], which can commonly be found in a variety of cereals and red and yellow fruits, have been identified as inhibitors of *H. pylori* HrsA.

#### 5.1.4. Inhibitors of *H. pylori* Cystathionine γ-Synthase (CGS)

CGS is a pyridoxal 5′-phosphate-dependent enzyme that catalyzes the initial step of the transsulfuration pathway converting L-cysteine to L-homocysteine in bacteria via a γ-replacement reaction, leading to the formation of cystathionine. Because of its absence in humans, this enzyme becomes an attractive drug target for antibacterials. Such enzymes have been identified and purified from *H. pylori* and hold great potential for anti-*H. pylori* drug development [42]. A couple of naphthopyranones, namely, 9-hydroxy-α-lapachone and α-lapachone [42], isolated from the stems of *Catalpa ovate* G. Don. and the wood of *Tabebuia heptaphylla* (Vell.) Mattos., respectively, have demonstrated substantial *H. pylori* CGS inhibition with considerable IC50 values. Another compound, juglone [43], which was isolated from the roots of *Juglans nigra* L. and *Juglans regia* L., was found to exhibit *H. pylori* CGS inhibition activity in vitro. A few lignans, including paulownin and yangambin [42], isolated from *Paulownia tomentosa* Steud and *Ocotea fasciculata* (Nees) Mez, respectively, have displayed significant inhibition of *H. pylori* CGS.

#### 5.1.5. Inhibitors of *H. pylori* Fatty Acid, Protein and Vitamin Biosynthesis

In addition to the above factors, which serve as excellent targets for *H. pylori* inhibitor design and drug development, other enzyme targets including malonyl-CoA:acyl carrier protein transacylase (FabD) and β-hydroxyacyl-ACP dehydratase (FabZ), inhibition of which has resulted in growth inhibition of *H. pylori*, have been less investigated. FabD and FabZ are key enzymes of the fatty acid synthesis pathway (FAS II) in *H. pylori*. The former confers the transfer of a malonyl moiety from malonyl-CoA to holo-ACP and forms malonyl-ACP, which acts as an elongation substrate for fatty acid synthesis, whereas FabZ is a primary dehydratase involved in the elongation cycles of unsaturated and saturated fatty acid synthesis [44]. Considering their importance and distribution, FabD and FabZ can be deemed a potential target for anti-*H. pylori* metabolites. An example FabD inhibitor is the natural compound juglone [43], which is also a CGS inhibitor. Another metabolite, emodin [44,45], a natural anthraquinone isolated from the rhizomes of *Rheum palmatum* and also a constituent of several traditional Chinese medicines, has been shown to inhibit FabZ in vitro with an IC50 of 9.7 μM, carrying enormous potential to be developed as an anti-*H. pylori* agent.

The biosynthesis of protein in bacteria commences on ribosomes with a formylated methionine (fMet), and successful removal of this group from the *N*-terminal end (deformylation) is essential to further the *N*-terminal processing of nascent polypeptides. Peptidyl deformylase (Pdf) cleaves the formyl group from the *N*-terminal, resulting in formic acid as a product of the reaction. Like any other bacteria, deformylation is also essential for the viability of *H. pylori* cells [46]. Selective inhibition of *H. pylori* Pdf may interrupt and impair the protein synthesis that, in turn, will stop and slow down cell growth and proliferation in *H. pylori* and is an important target for effective anti-*H. pylori* drug development. Caffeic acid phenethyl ester (CAPE) is so far the only known natural inhibitor of *H. pylori* Pdf [47]. CAPE is a principal component of honey bee propolis and has also been extracted from other plant parts. Paepalantine, an isocoumarin originally isolated from *Paepalanthus bromelioides* Silveira, demonstrated considerable inhibition of *H. pylori* by inhibiting membrane protein biosynthesis [48].

The futalosine pathway plays a vital role in menaquinone (vitamin K2) biosynthesis in *H. pylori*. Surprisingly, humans and beneficial bacteria from the human gut including Lactobacilli lack a futalosine pathway, which makes this pathway an appealing target for inhibitor design and development relating to *H. pylori* infection [49]. Siamycin I, a peptide antibiotic isolated from a culture filtrate of *Streptomyces* sp., has been shown to inhibit the futalosine pathway and prevent *H. pylori* colonization in gastric mucosa in mice. In addition, polyunsaturated fatty acids such as eicosapentaenoic acid (EPA) and docosahexaenoic acid (DHA), for which microalgae are regarded as an excellent source, were also reported to inhibit *H. pylori* in the same experimental model [50,51].

#### 5.1.6. Inhibitors of *H. pylori* Biofilm Formation

Bacterial biofilms formed by aggregated colonies shielded by an extracellular matrix and attached to a surface are a critical part of environmental adaptability and infection. Several studies have demonstrated that *H. pylori* also forms biofilms in the environment, on abiotic surfaces in vitro and on the mucosal epithelium in the human stomach [52].

Biofilm formation on the gastric mucosa provides opportunities for secret virulence factors which fortify host–pathogen interactions to evade the host’s innate defense system, and neutralizes the actions of combinatorial antibiotic treatments and endogenous antimicrobial peptides as well. In addition, antibiotic resistance mutations are more frequently generated in the biofilm [53]. Summarizing the above facts, biofilm formation in *H. pylori* is one of the major possible reasons for eradication failure, which emphasizes the need to hunt for safe and effective biofilm-inhibiting agents. In this regard, phillygenin [54] and armeniaspirol A [55], isolated from the leaves of *Forsythia suspensa* and culture filtrates of *Streptomyces armeniacus*, respectively, demonstrated strong in vitro biofilm inhibition in *H. pylori*. However, seeing that this area has potential for the control and management of H. pylori infection, more biofilm-inhibiting agents are warranted in future.

**Figure 2:**
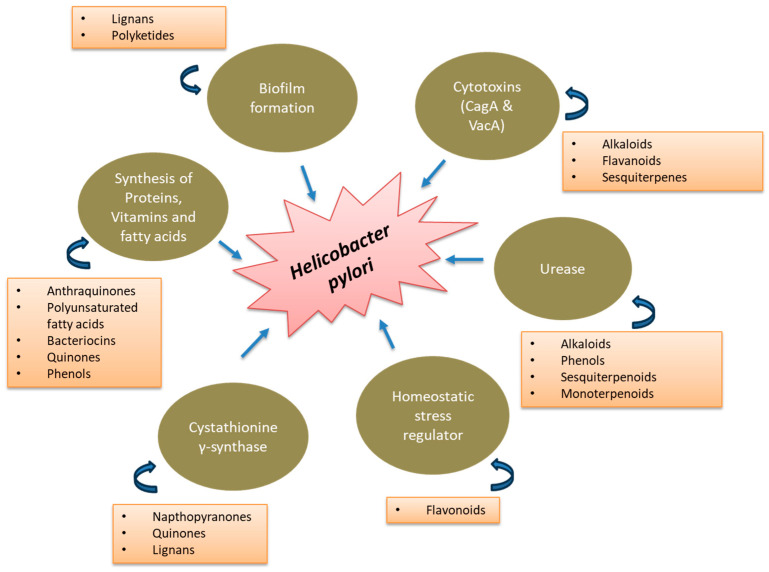
A graphical summary of the different mechanisms of action of *H. pylori* inhibition of different classes of plant and microbial natural compounds.

**Figure 3:**
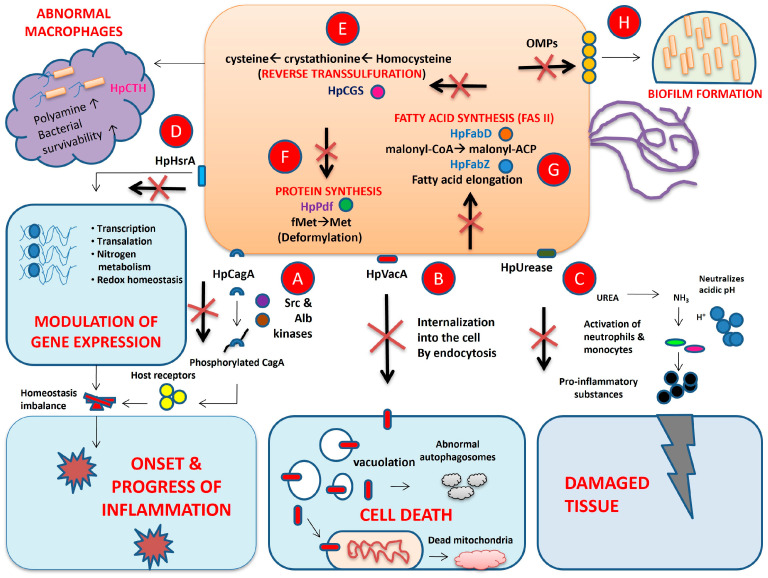
Schematic representation of inhibition mechanisms reported for plant and microbial natural compounds against *H. pylori* (Hp). A. Inhibition of HpCagA (cytotoxin-associated gene A), B. HpVacA (vacuolating cytotoxin A), C. Urease, D. HpHsrA (homeostatic stress regulator A), E. HpCGS (cystathionine γ-synthase), F. HpPdf (peptide deformylase), G. HpFabD (malonyl-CoA:acyl carrier protein transacylase) and HpFabZ (β-hydroxyacyl-ACP dehydratase) and H. biofilm formation.

The details of these metabolites, including the isolation source, mechanism of action and bibliographic links, are presented in Table 1. A graphical summary of *H. pylori* inhibition by different classes of compounds (Figure 2) and a pictorial representation of the action mechanisms demonstrated by these metabolites have been provided to better the understanding of readers (Figure 3). Further, representative structures with the exact mechanism of action discussed above are reported in Figure S1.

### 5.2. Inhibitors of H. pylori with No Specific Mechanism of Action

Thus far, the natural metabolites with reported particular mechanisms of action which interfere in the cellular processes of or inhibit the virulence factors of *H. pylori* have been reviewed. A major part of natural compounds displayed in vitro growth inhibition of *H. pylori*; nevertheless, it has not been investigated for an exact mechanism of action and is reviewed in this section (Table 2). Naphthoquinones isolated from root extract of *Reynoutria japonica*, such as 2-ethoxy-6-acetyl-7-methyljuglone, 2-methoxy-6-acetyl-7-methyljuglone, 2-methoxy-7-acetonyljuglone and 3-acetyl-7-methoxy-2-methyljuglone, demonstrated significant in vitro growth inhibition against *H. pylori* [56]. A couple of phenol glycosides, 4,6-dihydroxy-2-methoxyphenyl-1-*O*-*β*-D-glucopyranoside and 4-hydroxy-2,6-dimethoxyphenyl-1-*O*-α-L-rhamnopyranosyl (1-6)-β-D-glucopyranoside, isolated from the organic extract of *Hypericum erectum* displayed considerable anti-*H. pylori* activity [57]. A few sesquiterpenoids, namely, (*Z*)-lanceol, (*Z*)-α-santalol and (*Z*)-*β*-santalol, isolated from the heartwood of *Santalum album* were able to inhibit *H. pylori* growth in vitro with a promising IC50 [58]. Fraxetin, syringic acid, (1*S*,2*R*)-1,2-bis(4-hydroxy-3-methoxyphenyl)-1,3-propanediol and (2*R*,3*S*)-2-ethoxychroman-3,5,7-triol-7-*O*-β-D-apiofuranoside from the root bark of *Ulmus davidiana* var. *japonica* demonstrated significant anti-*H. pylori* activity in vitro [59]. Additionally, heterophylliin G, nobotanin B, procyanidin B-5, strictinin [60], allicin, allyl-methyl thiosulfinate [61], berberine, dehydrocorydaline [62], cinnamaldehyde [63], eldaricoxide A, manoyl oxide acid [64], ethyl galbanate, sanandajin [65], eugenol [66], myricetin-3-*O*-β-D-glucuronide, quercetin-3-*O*-β-D-galactopyranoside-6″-gallate, tiliroside [67] and olean-12-en-3-one [68], which showed in vitro/in vivo *H. pylori* activity, have been isolated from diverse plants or plant parts.

Though few in number, bacterial metabolites have also been explored as latent anti- *H. pylori* agents. Strong inhibition of *H. pylori* growth was reported for CJ-13,136, an alkaloid isolated from the bacterium *Pseudonocardia* sp. [69]. Similarly, bacteriocins lacticin A164 and BH5 isolated from *Lactococcus lactis* demonstrated promising anti-*H. pylori* activity and can be explored as probable antibacterials [70].

Fungal metabolites, including two polyketides, namely, (2*E*)-1-[(5-hydroxy-7-methoxy-2-methyl-4-oxo-4*H*-1-benzopyran-3-yl)methyl]3-methyl-2-pentenedioate and (2*S*,3*S*)-5-hydroxy-3-hydroxymethyl-7-methoxy-2-methyl-4-chromanone, isolated from *Trichoderma* sp. showed potent growth inhibition at an IC50 range of 2–8 µg/mL [71]. Two sterols, 3β,5α,6β-trihydroxyergosta-7,22-diene and ergosterol, and two benzophenones, monomethylsulochrin and rhizoctonic acid, isolated from an endophytic fungus *Rhizoctonia* sp. were reported to have anti-*H. pylori* activities [72]. Ergosterol and monomethylsulochrin were also isolated from *Aspergillus* sp., reportedly an endophytic fungus from *Cynodon dactylon*. Additionally, 3*β*-hydroxy-5*α*,8*α*-epidioxy-ergosta-6,22-diene and helvolic acid demonstrating considerable inhibition of *H. pylori* were isolated from the same endophytic isolate [73]. A few Bis-naphtho[2,3-b]pyrones, including aurasperone A, B and F, and asperpyrone A with growth-inhibiting activity against *H. pylori* were reported from the filamentous fungus *Aspergillus* sp. [74]. Demethylincisterol A3, an ergosterol derivative showing moderate growth inhibition of the pathogen, was isolated from fruiting bodies of the mushroom *Daedaleopsis confragosa* [75]. Two fatty acids, (9*E*)-11-oxo-9-octadecenoic acid and (9*E*)-methyl ester 9-octadecenoic acid, with reasonable anti-*H. pylori* potential were isolated from the fruiting bodies of *Amanita hemibapha* subsp*. javanica* [76].

**Table 2:** Details of plant and microbial metabolites discovered to have potential anti-*H. pylori* activity without any specific mechanism of action.

Sl No.	Name of the Compounds	Chemical Classes	Sources of Isolation	Experimental Evidence	Dosage (MIC/IC50/%Inhibition)	References
1.	(1*S*,2*R*)-1,2-Bis(4-hydroxy-3-methoxyphenyl)-1,3-propanediol	Phenols	Root bark of *Ulmus davidiana* var. *Japonica* (Sarg. ex Rehder) Nakai	In vitro	8–16 µg/mL	[57]
2.	(2*E*)-1-[(5-Hydroxy-7-methoxy-2-methyl-4-oxo-4*H*-1-benzopyran-3-yl)methyl]3-methyl-2-pentenedioate	Polyketides	culture filtrate of *Trichoderma* sp.	In vitro	2–8 µg/mL	[71]
3.	(2*R*,3*S*)-2-Ethoxychroman-3,5,7-triol-7-*O*-β-D-apiofuranoside	Chromane derivatives	Root bark of *Ulmus davidiana* var. *japonica* (Sarg. ex Rehder) Nakai	In vitro	10.5–21.2 µg/mL	[57]
4.	(2*S*,3*S*)-5-Hydroxy-3-hydroxymethyl-7-methoxy-2-methyl-4-chromanone	Polyketides	culture filtrate of *Trichoderma* sp.	In vitro	2–8 µg/mL	[71]
5.	(9*E*)-11-Oxo-9-octadecenoic acid	Fatty acids	Fruiting bodies of *Amanita hemibapha* subsp. *javanica*	In vitro	38% inhibition	[76]
6.	(9*E*)-Methyl ester 9-octadecenoic acid	Fatty acids	Fruiting bodies of *Amanita hemibapha* subsp. *javanica*	In vitro	80.5% inhibition	[76]
7.	(*Z*)-Lanceol	Sesquiterpenoids	Heartwood of *Santalum albumi* L.	In vitro	31.3–125 µg/mL	[56]
8.	(*Z*)-α-Santalol	Sesquiterpenoids	Heartwood of *Santalum album* L.	In vitro	7.8–31.3 µg/mL	[56]
9.	(*Z*)-*β*-Santalol	Sesquiterpenoids	Heartwood of *Santalum album* L.	In vitro	7.8–31.3 µg/mL	[56]
10.	2-Ethoxy-6-acetyl-7-methyljuglone	Naphthoquinones	Root extract of *Reynoutria japonica* (Houtt.)	In vitro	0.04–0.08 µg/mL	[52]
11.	2-Methoxy-6-acetyl-7-methyljuglone	Naphthoquinones	Root extract of *Reynoutria japonica* (Houtt.)	In vitro	0.05–0.07 µg/mL	[52]
12.	2-Methoxy-7-acetonyljuglone	Naphthoquinones	Root extract of *Reynoutria japonica* (Houtt.)	In vitro	0.02–0.13 µg/mL	[52]
13.	3-Acetyl-7-methoxy-2-methyljuglone	Naphthoquinones	Root extract of *Reynoutria japonica* (Houtt.)	In vitro	2.59–8.58 µg/mL	[52]
14.	3β,5α,6β-Trihydroxyergosta-7,22-diene	Sterols	Culture filtrates of *Rhizoctonia* sp.	In vitro	25 µg/mL	[72]
15.	3β-Hydroxy-5α,8α-epidioxy- ergosta-6,22-diene	Sterols	Culture filtrates of *Aspergillus* sp.	In vitro	30 µg/mL	[73]
16.	4,6-Dihydroxy-2-methoxyphenyl-1-*O*-β-D-glucopyranoside	Phenol glycosides	*Hypericum* *Erectum* Thunberg	In vitro	7.3 μg/mL	[53]
17.	4-Hydroxy-2,6-dimethoxyphenyl-1-*O*-α-L-rhamnopyranosyl(1-6)-β-D-glucopyranoside	Phenol glycosides	*Hypericum* *Erectum* Thunberg	In vitro	27.3 μg/mL	[53]
18.	Allicin	Thiosulfinic acid esters	*Allium sativum* L.	In vitro	16 µg/mL	[59]
19.	Allyl-methyl thiosulfinate	Alkanethiosulfinic acid esters	*Allium sativum* L.	In vitro	24 µg/mL	[59]
20.	Asperpyrone A	Bis-naphtho[2,3-b]pyrones	Culture filtrates of *Aspergillus* sp.	In vitro	4 μg/mL	[74]
21.	Aurasperone A	Bis-naphtho[2,3-b]pyrones	Culture filtrates of *Aspergillus* sp.	In vitro	8–16 μg/mL	[74]
22.	Aurasperone B	Bis-naphtho[2,3-b]pyrones	Culture filtrates of *Aspergillus* sp.	In vitro	8–16 μg/mL	[74]
23.	Aurasperone F	Bis-naphtho[2,3-b]pyrones	Culture filtrates of *Aspergillus* sp.	In vitro	4 μg/mL	[74]
24.	Berberine	Alkaloids	Dried tubers of *Corydalis yanhusuo* W.T. Wang	In vitro	25 μg/mL	[60]
25.	Cinnamaldehyde	Phenylpropanoids	*Cinnamomum cassia* (L.) J. Presl	In vitro	2 μg/mL	[61]
26.	CJ-13,136	Alkaloids	Culture filtrates of *Pseudonocardia* sp.	In vitro	0.0001 μg/mL	[69]
27.	Dehydrocorydaline	Alkaloids	Dried tubers of *Corydalis yanhusuo* W.T. Wang	In vitro	12.5 μg/mL	[60]
28.	Demethylincisterol A3	Ergosterol derivatives	Fruiting bodies of *Daedaleopsis confragosa*	In vitro	33.9% inhibition	[75]
29.	Eldaricoxide A	Diterpenoids	Needles of *Pinus eldarica* Medw.	In vitro	29.49 μg/mL	[62]
30.	Ergosterol	Sterols	Culture filtrates of *Rhizoctonia* sp. and *Aspergillus* sp.	In vitro	20–30 µg/mL	[72,73]
31.	Ethyl galbanate	Sesquiterpene coumarins	Roots of *Ferula pseudalliacea* Rech.f.	In vitro	64 μg/ml	[63]
32.	Eugenol	Phenols	Clove oil	In vitro	2 μg/mL	[64]
33.	Fraxetin	Coumarins	Root bark of *Ulmus davidiana* var. *japonica* (Rehder) Nakai.	In vitro	5.2–10.40 μg/mL	[57]
34.	Helvolic acid	Steroids	Culture filtrates of *Aspergillus* sp.	In vitro	8 µg/mL	[73]
35.	Heterophylliin G	Tannins	*Corylus heterophylla* Fisch. ex Trautv.	In vitro	12.25–25 µg/mL	[58]
36.	Lacticin A164	Bacteriocins	Culture filtrates of *Lactococcus lactis*	In vitro	0.097–0.390 µg/mL	[70]
37.	Lacticin BH5	Bacteriocins	Culture filtrates of *Lactococcus lactis*	In vitro	0.097–0.390 µg/mL	[70]
38.	Manoyl oxide acid	Diterpenoids	Needles of *Pinus eldarica* Medw.	In vitro	26.72 μg/mL	[62]
39.	Monomethylsulochrin	Benzophenones	Culture filtrates of *Rhizoctonia* sp. and *Aspergillus* sp.	In vitro	10 µg/mL	[72,73]
40.	Myricetin-3-*O*-β-D-glucuronide	Phenols	*Potentilla* spp.	In silico	--	[65]
41.	Nobotanin B	Tannins	*Melastoma candidum* D.Don	In vitro	12.25–25 µg/mL	[58]
42.	Olean-12-en-3-one	Triterpenoids	Figs of *Ficus vallis-choudae* Delile	In vitro	6.1–10.4 µg/mL	[66]
43.	Procyanidin B-5	Tannins	*Vitis vinifera* L.	In vitro	25–50 µg/mL	[58]
44.	Quercetin-3-*O*-β-D-galactopyranoside-6″-gallate	Phenols	*Potentilla* spp.	In silico	--	[65]
45.	Rhizoctonic acid	Benzophenones	Culture filtrates of *Rhizoctonia* sp.	In vitro	25 µg/mL	[72]
46.	Sanandajin	Disesquiterpene coumarins	Roots of *Ferula pseudalliacea* Boiss	In vitro	64 μg/mL	[63]
47.	Strictinin	Tannins	*Elaeagnus umbellate* Thunb.	In vitro	6.25–25 µg/mL	[58]
48.	Syringic acid	Phenols	Root barks of *Ulmus davidiana* var. *japonica* (Rehder) Nakai.	In vitro	4.95–9.90 µg/mL	[57]
49.	Tiliroside	Phenols	*Potentilla* spp.	In silico	--	[65]

## 6. Cytotoxicity as a Challenge in Anti-*H. pylori* Drug Development

The cytotoxicity of a chemical compound, whether natural or synthetic, is considered to be a dose-dependent outcome and is a key deliberation in the safety assessment of any compound and a major hurdle in therapeutics development. Many natural compounds, whether derived from a plant, bacteria or fungi, can be cytotoxic, depending on their chemical structure, the context of use and the administered doses. In this regard, the goal of anti-*H. pylori* drug development must focus on managing the dosage to minimize harm to healthy cells and tissues. Herein, we attempt to review the known cytotoxicity or pharmacokinetics of some of the potent anti-*H. pylori* compounds. While no specific data are available on the in vivo pharmacokinetics, most studies have focused on isolation, structure elucidation and in vitro antibacterial activity. However, few compounds have been reported to have negligible toxicity to the normal cells in in vivo models. For example, armeniaspirol A, a potent anti-*H. pylori* metabolite isolated from culture filtrates of *Streptomyces armeniacus*, was found to have negligible toxicity at effective therapeutic doses. Further, when administered at 10 times the effective dosage for five consecutive days, no serious adverse effects were detected. Similarly, Siamycin I also reportedly exhibits negligible toxicity to mammalian cells. Bis-naphtho[2,3-b]pyrones, which appear to be potent antibacterials, among others, have been reported to have negligible or no toxicity against normal mammalian cells at biologically active concentrations [77,78,79]. There are no reports on the cytotoxicity of naphthoquinones reviewed in this study; however, juglone is reported to exhibit toxicity to normal fibroblast cells in particular. Its toxicity can be detrimental to other organisms as it is used as a herbicide and fish toxin [80].

## 7. Recent Approaches to Improve the Bioavailability and Efficacy of Natural Anti-*H. pylori* Agents

The unprecedented surge in the global prevalence of *H. pylori* infection has become a serious concern and is associated with rising numbers of cases of antimicrobial resistance worldwide. Further, the ability of the bacterium to form biofilms can be significantly correlated to the crisis relating to the rise of drug resistance rates. Secondly, the protective function of the mucus layer covering the gastrointestinal epithelium prevents anti-*H. pylori* drugs reaching the target sites, resulting in low drug bioavailability, provided that adequate drug bioavailability must be reached to mitigate the infection. To overcome these setbacks, new-generation antibiotics such as nanoparticle-based antimicrobials, including silver nanoparticles [AgNPs], gold nanoparticles [AuNPs] and zinc oxide nanoparticles [ZnONPs], are being realized as novel strategies to control *H. pylori* infection [81]. In addition, nanotechnology-driven drug delivery systems are being rapidly developed, ensuring features such as mucus penetration, precisive targeting and stimuli-responsive measured drug release [82]. This approach is reported to enhance the bioavailability and efficiencies of orally administered anti-*H. pylori* drugs; moreover, it diminishes the side effects of the original drugs and provides better benefits to patients. Other approaches, such as enclosing the candidate drug inside a defensive nanoshell (encapsulation), protect and enhance its solubility and stability and reduce side effects. Nanoparticles, nanoemulsions and various polymer-based systems are some of the key methods used for nanoencapsulation approaches. Similarly, hybrid drug delivery strategies which combine different systems (materials) to create a new system with synergistic properties from both systems accomplish better control over drug release and improved stability and targeting accuracy. Examples include lipid–polymer nanoparticles and combinations of different manufacturing processes such as compression and 3D printing.

## 8. Discussion and Conclusions

The entrepreneurs associated with the commercialization of natural products at present are showing huge interest in natural product-based medicines. A broad range of bioactive products have been derived from natural sources including plants and microorganisms. Be it the recent anti-malarial drug ‘artemisin’ developed from the plant *Artemisia annua*, the antibiotic ‘streptomycin’ produced from *Streptomyces griseus* or the much-needed anticancer drug ‘taxol’ produced from the endophytic fungus *Fusarium solani*, in every way natural products continue to offer the pharmaceutical industry enormous opportunities to develop new and potent drugs against new and emerging diseases. Bioassay-guided fractionation and purification have demonstrated successful isolation and identification of active natural compounds from a mixture of compounds prepared from plant extracts or microbial culture filtrates. Alternatively, bioinformatics approaches such as subtractive proteomics, virtual screening and molecular docking coupled with dynamic simulation studies are contributing to identifying potential drugs or inhibitors, thereby reducing both the time and cost associated with traditional pharmaceuticals development.

The natural compounds reviewed herein display massive inhibitory potential against *H. pylori*. Overall, quinones and quinone derivatives (including anthraquinones and naphthoquinones) and polyketides (including bis-naphtho[2,3-b]pyrones) appear to be the most promising antimicrobials. The biological activity of quinones can be attributed to the chemical structure and the position of substituents. In most cases, quinones act as intercalating agents in the DNA double helix, which is responsible for their antibacterial activity [83]. Moreover, the polarity of substituents is a central factor in determining the antibacterial activity, as in case of anthraquinones and napthoquinones; the greater the polarity, the activity increases [84]. Antibacterial activity in polyketides is also linked to their specific chemical structures. Most polyketides act as inhibitors of protein and nucleic acid synthesis and cell membrane disruption, while some may act as DNA akylation agents, thereby inhibiting the bacterial growth [85]. Bis-naphtho[2,3-b]pyrones, a type of aromatic polyketide, have demonstrated potent anti-*H. pylori* activity. They have a free hydroxyl group at the C-8 position which is believed to be crucial for their activity [86]. Bacteriocins (isolated from culture filtrates of *Lactococcus lactis*) were among others with significant potential as *H. pylori* inhibitors. Bacteriocins are amphipathic, with discrete hydrophobic and hydrophilic faces that enable their contacts with bacterial membrane-bound protein targets and can disrupt the membrane structure and functions [87]. Similarly, CJ-13,136, a quinoline alkaloid derived from the culture filtrate of *Pseudonocardia* sp., was reported to have strong inhibition against the bacterium. The exact structure–activity relationship is not known, though it can be hypothesized that the alkaloid skeleton might play a role.

Thus far, the literature cited in this review has exploited virulence factors such as CagA, VacA and urease as major targets for antibacterial discovery. However, the discovery of natural compounds targeting disruption of the cell wall and cell membrane, explicitly the enzymes and proteins involved in peptidoglycan synthesis (as in the case of beta lactams and vancomycin), is highly lacking and can be considered an important drawback in anti-*H. pylori* drug development. Further, peptidoglycan is a very essential bacterial component, absent in mammalian cells, which offers a way to be able to selectively kill the bacterium without causing much harm to the human cells. Further, the experimentation used for assessing the antibacterial activity was mostly in vitro, which is confined to artificial and controlled environments only. Though a few studies went further, to in vivo models, the toxicity parameters remain unexplored. Therefore, a detailed investigation of animal models and clinical trials is warranted to prove the efficacy and safety profiles of these metabolites. Thus, the dearth of in vivo evidence involving a whole living organism is a significant obstacle on their path to becoming a lead candidate.

Unarguably, medicinal plants are the largest contributors of anti-*H. pylori* metabolites, and the majority of the reported metabolites are of plant origin. The microbial metabolites have been gaining particular interest as a reservoir of structurally and functionally diverse antimicrobial metabolites. Microbial metabolites, which are being used as antibiotic drugs, include cephalosporins (from the fungus *Acremonium chrysogenum*), tetracyclines (from the bacterium *Streptomyces aureofaciens*), erythromycins (from the bacterium *Saccharopolyspora erythraea*) and neomycin (from the bacterium *Streptomyces fradiae*). The use of microbes facilitates economic, large-scale production of the metabolite of interest. Further, microbial production can be more eco-friendly than synthetic procedures and can use renewable feedstocks as nutrient substrates, thereby reducing/utilizing wastes. Moreover, modulation of biosynthetic pathways of interest through precision fermentation using recombinant DNA technology can improve the yield of a desired product. Further, routine screening of microbial metabolites will introduce novel metabolites with potent activity against *H. pylori*. Despite such tremendous advantages, microbial resources have been underestimated while discovering natural inhibitors of *H. pylori*.

Despite continued demand for natural product-based therapeutics, certain limitations are major obstacles in their translation to marketed drugs. Insignificant pharmacokinetics (absorption, distribution, metabolism, elimination) and toxicity profiles associated with the isolated natural compounds are a major cause of failure in the preclinical and clinical stages, and can be considered a major limitation in natural product-based drug discovery. Another limitation is that there are certain *H. pylori* targets that cannot be influenced by natural compounds, such as efflux pumps, which bacteria use to expel toxic compounds, including antibiotics. This general mechanism of resistance can be a major obstacle, and cannot be undone unless treated with an efflux pump inhibitor along with natural antimicrobials. In conclusion, we insist that research must be focused on bioassay-guided fractionation and purification strategies to isolate novel anti-*H. pylori* metabolites from unique resources including unexplored plant species and microbial niches. Further, planning must also be brought into in vivo experiments and preclinical and clinical trials. Moreover, research must be directed to investigate the synergistic effects of two or more potent natural compounds and must even combine a natural inhibitor with previously used synthetic drugs (for example, amoxicillin, clarithromycin or metronidazole) with the aim of achieving effective management and successful eradication of *H. pylori* infection. Above all, we hope that, in the coming days, more natural products will be explored for their ability to inhibit *H. pylori* and adequate efforts made to develop new and effective antibiotics to overcome the drug resistance in *H. pylori*.

## Figures and Tables

**Table 1:** Details of plant and microbial metabolites discovered to exhibit potential anti-*H. pylori* activity with a specific mechanism of action.

Sl No.	Name of the Compounds	Chemical Classes	Sources of Isolation	Experimental Evidence	Dosage (MIC/IC50/%Inhibition)	Remarks	References
Inhibitors of *H. pylori* cytotoxins							
1.	Evodiamine	Alkaloids	Fruits of *Evodia rutaecarpa* (Juss.) Benth.	In vitro	1.5–24.2 μg/mL	Down-regulation of urease and diminished translocation of CagA and VacA Down-regulation of gene expressions of replication and transcription machineries	[26]
2.	Hesperetin	Flavonoids	Citrus fruits	In vitro	4–8 μg/mL	Down-regulation of virulence gene expressions	[27,39]
3.	β-Caryophyllene	Sesquiterpenes	Essential oil of *Commiphora gileadensis* (L.) C. Chr.	In vitro	1000 µg/mL	Growth inhibition, down-regulation of virulence gene expressions	[28]
4.	Thymol	Monoterpenoids	*Nigella sativa* L.	In silico	--	CagA and VacA inhibition	[30]
5.	Thymoquinone	Monoterpenoids	*Nigella sativa* L.	In silico	--	CagA and VacA inhibition	[30]
**Inhibitors of *H. pylori* urease**							
6.	Chlorogenic acid	Phenols	Gel from *Aloe vera* (L.) Burm. f.	In silico	--	--	[36]
7.	Diosmin	Flavonoids	Citrus fruits	In silico	--	--	[37]
8.	Methyl rosmarinate	Phenols	Stem bark of *Cordia Africana* Lam.	In vitro and in silico	31.25 μg/mL	--	[32]
9.	Pyrocatechol	Phenols	*Aloe vera* (L.) Burm. f.	In silico	--	--	[36]
10.	Sanguinarine	Alkaloids	*Zanthoxylum nitidum* (Roxb.) DC.	In vitro	159.5 μg/mL	--	[33]
11.	Terpineol	Monoterpenoids	Widely found in flowers like narcissus and freesia, in herbs including marjoram, oregano and rosemary and in lemon peel oil	In vitro and in silico	1.443 μg/mL	--	[34]
12.	Zerumbone	Sesquiterpenoids	*Zingiber zerumbet* (L.) Roscoe ex Sm	In vitro	10.91 μg/mL		[35]
**Inhibitors of *H. pylori* homeostatic stress regulator (HsrA)**							
13.	Apigenin	Flavonoids	Widely present in cereals and red and yellow fruits	In vitro	8 μg/mL	--	[39]
14.	Chrysin	Flavonoids	Widely present in cereals and red and yellow fruits	In vitro	4–8 μg/mL	--	[39]
15.	Kaempferol	Flavonoids	Widely present in cereals and red and yellow fruits	In vitro	4–8 μg/mL	--	[39]
**Inhibitors of *H. pylori* cystathionine γ-synthase (CGS)**							
16.	9-Hydroxy-α-lapachone	Naphthopyranones	Stems of *Catalpa ovata* G. Don.	In vitro	2.32 µg/mL	--	[42]
17.	α-Lapachone	Naphthopyranones	Wood of *Tabebuia heptaphylla* (Vell.) Mattos.	In vitro	2.66 µg/mL	--	[42]
18.	Juglone	Quinones	Roots of *Juglans nigra* L. and *Juglans regia* L.	In vitro	1.21 µg/mL	--	[42]
19.	Paulownin	Lignans	*Paulownia tomentosa* Steud.	In vitro	7.03 µg/mL	--	[42]
20.	Yangambin	Lignans	*Ocotea fasciculata* (Nees) Mez.	In vitro	12.05 µg/mL		[42]
**Inhibitors of *H. pylori* fatty acid, protein and vitamin biosynthesis**							
21.	Emodin	Anthraquinones	Rhizomes of *Rheum palmatum* L. and other traditional Chinese medicines	In vitro	2.6 μg/mL	β-hydroxyacyl-ACP dehydratase (FabZ) inhibition	[45]
22.	Caffeic acid phenethyl ester	Phenols	Honey bee propolis	In vitro	1.14 μg/mL	Peptide deformylase (pdf) inhibition	[47]
23.	Paepalantine	Isocoumarins	Capitula of *Paepalanthus bromelioides* Silveira	In vitro and in silico	128 μg/mL	Inhibiting membrane protein synthesis	[48]
24.	Siamycin I	Bacteriocins	Culture filtrates of *Streptomyces* sp.	In vitro and in vivo	5.4 μg/mL (*H. pylori* colonization was reduced by 68% in vivo)	Inhibition of futalosine pathway of melaquinone (vitamin K2) biosynthesis	[49]
25.	Docosahexaenoic acid	Polyunsaturated fatty acids	Culture filtrates of *Schizochytrium* sp.	In vitro and in vivo	32.8 μg/mL (*H. pylori* colonization was reduced by 78% in vivo)	Inhibition of futalosine pathway of melaquinone (vitamin K2) biosynthesis	[49]
26.	Eicosapentaenoic acid	Polyunsaturated fatty acids	Culture filtrates of *Phaeodactylum tricornutum*	In vitro and in vivo	30.2 μg/mL (*H. pylori* colonization was reduced by 96% in vivo)	Inhibition of futalosine pathway of melaquinone (vitamin K2) biosynthesis	[49]
27.	Juglone	Quinones	Roots of *Juglans nigra* L. and *Juglans regia* L.	In vitro	3.48 and 5.22 µg/mL	Inhibition of malonyl-CoA:acyl carrier protein transacylase (FabD) and β-hydroxyacyl-ACP dehydratase (FabZ)	[42]
**Inhibition of biofilm formation in *H. pylori***							
28.	Phillygenin	Lignans	Leaves of *Forsythia suspensa* (Thunb.) Vahl.	In vitro	16–64 μg/mL	Biofilm inhibition	[54]
29.	Armeniaspirol A	Polyketides	Culture filtrates of *Streptomyces armeniacus*	In vivo	4–16 μg/mL	Biofilm inhibition	[55]

## Data Availability

The original contributions presented in this study are included in the article/Supplementary Material. Further inquiries can be directed to the corresponding authors.

## Supplementary Materials

The following supporting information can be downloaded at https://www.mdpi.com/article/10.3390/biotech14040094/s1, Figure S1. Chemical structures of alkaloids, flavonoids, terpenes, terpenoids, phenols, naphtopyranones, quinones, lignans, anthraquinones and isocumarins with potential anti-*H. pylori* activity with specific mechanisms of action.
