# Supplementary material for: A Comprehensive Review of Plant and Microbial Natural Compounds as Sources of Potential Helicobacter pylori-Inhibiting Agents
Source: BioTech (Basel). 2025 Nov 26;14(4):94. doi: 10.3390/biotech14040094 (PMC12730657; doi:10.3390/biotech14040094)
Supplement: Supplementary file 1 [file biotech-14-00094-s001.zip › biotech-3961226-supplementary.pdf]

BioTech

Review

A Comprehensive Review of Plant and Microbial Natural Compounds as Sources of Potential *Helicobacter pylori*-Inhibiting Agents

Srichandan Padhi [1,*], Swati Sharma [2], Puja Sarkar [3], Marco Masi [4,*], Alessio Cimmino [4] and Amit Kumar Rai [3,5*]

[1] University Centre for Research and Development, Chandigarh University, Mohali 140413, India
[2] Advanced Centre of Research and Innovation, Chandigarh Group of Colleges Jhanjeri, Mohali 140307, India; swatishimla12@gmail.com
[3] Food and Nutrition Biotechnology, BRIC-National Agri-Food and Biomanufacturing Institute (NABI), Mohali 140306, India; pujas8981@gmail.com
[4] Department of Chemical Sciences, University of Naples Federico II, Complesso Universitario Monte S. Angelo, Via Cintia, 80126 Naples, Italy; alessio.cimmino@unina.it
[5] Department of Botany, University of Delhi, New Delhi- 110007, India; amitraikvs@gmail.com
* Correspondence: srichandan.e19154@cumail.in (S.P.); marco.masi@unina.it (M.M.); amit.rai@nabi.res.in (A.K.R.)

Contents

Figure S1. Chemical structures of alkaloids, flavonoids, terpenes, terpenoids, phenols, naphtopyranones, quinones, lignans, anthraquinones and isocumarins having potential anti- *H. pylori* activity with specific mechanisms of action.

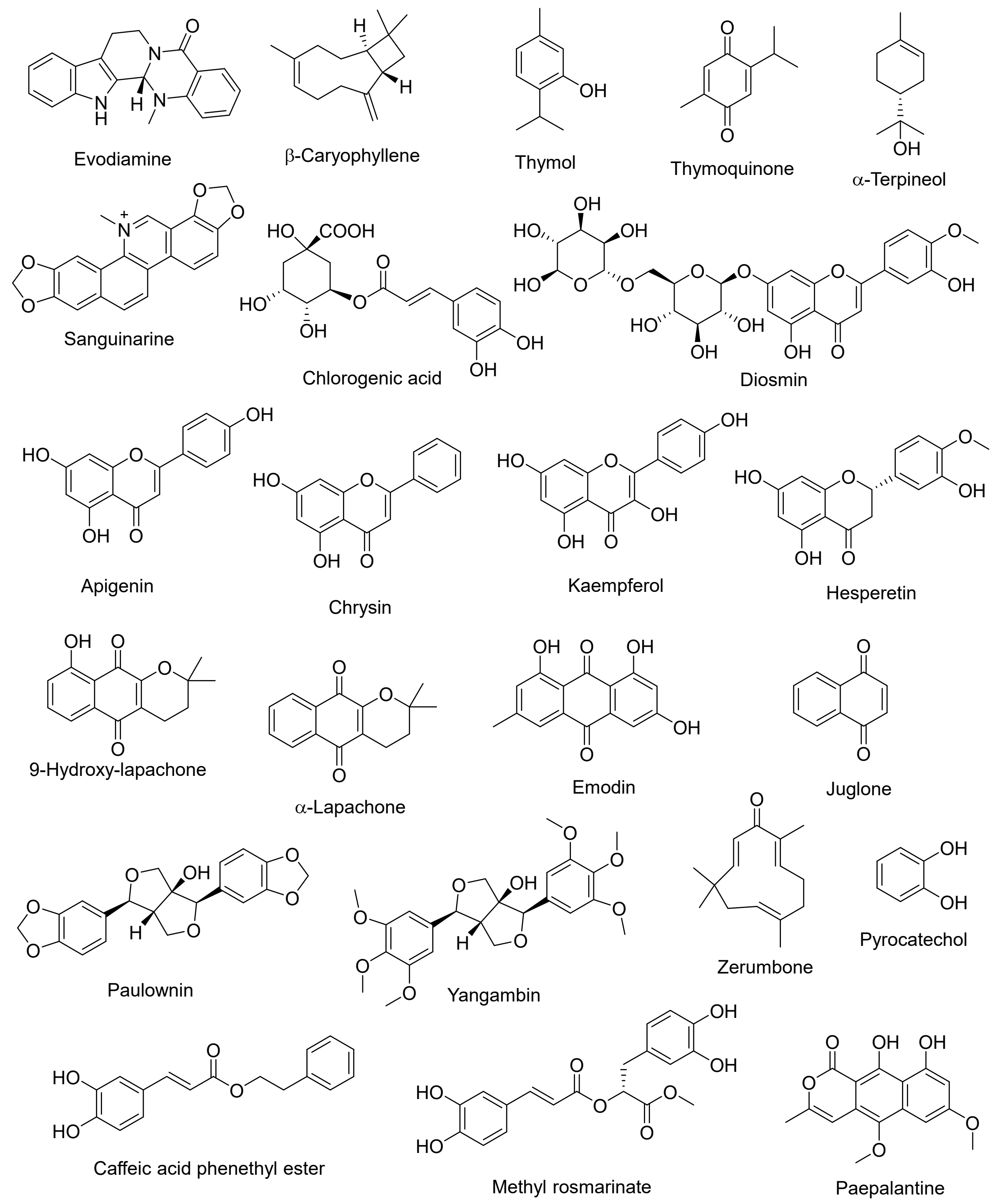

Figure S1. Chemical structures of alkaloids, flavonoids, terpenes, terpenoids, phenols, naphtopyranones, quinones, lignans, anthraquinones and isocumarins having potential anti- *H. pylori* activity with specific mechanisms of action.
